# TransTACs: novel heterobispecific antibodies for targeted protein degradation in cancer therapy

**DOI:** 10.1038/s41392-024-02062-1

**Published:** 2024-11-26

**Authors:** Wolfgang Walther, Sebastian Torke, Ulrike Stein

**Affiliations:** 1 Experimental and Clinical Research Center, Charité - Universitätsmedizin Berlin and Max-Delbrück-Center for Molecular Medicine, Berlin, Germany; 2 German Cancer Consortium (DKTK), Berlin and German Cancer Research Center (DKFZ), Heidelberg, Germany

**Keywords:** Drug development, Biologics

In a recent article in *Nature*, Dingpeng Zhang and colleagues^1^ describe a novel approach for the degradation of target proteins. Their newly designed heterobispecific antibody modality hijacks the transferrin receptor internalization machinery on one hand and simultaneously marks a protein of interest (POI) for lysosomal degradation on the other. This structure therein constitutes a promising innovative addition to the rapidly evolving field of extracellular protein degradation in cancer drug development.

Successful cancer therapy relies on the identification of targets representing key molecules to drive cancer growth, progression, invasion and migration. Having such targets at hand, intervention strategies can be developed to specifically inhibit their action. Such strategies often use small molecules to intervene in target gene expression, directly acting on target function or interrupting target associated signaling pathways.^2^ Alternative strategies aim at the entire elimination of the target proteins via proteolytic degradation for effective tumor inhibition. In this regard, multiple technologies were developed for the targeting of intracellular as well as for extracellular proteins, using proteolysis-targeting chimeras (PROTACs), proteolysis targeting antibodies (PROTABs) or other target protein degradation (TPD) means, to guide the POI towards either proteasomal or lysosomal degradation.^3^ The attractiveness to fully eliminate cancer promoting/driving target proteins—with the aim to stop tumor growth or to eliminate cancer cells—has led to an explosive growth of this research field in the last decade. The current research article by Dingpeng Zhang and colleagues describes a promising new approach with increased effectiveness of POI degradation and favorable cancer cell specificity.

**Fig. 1:**
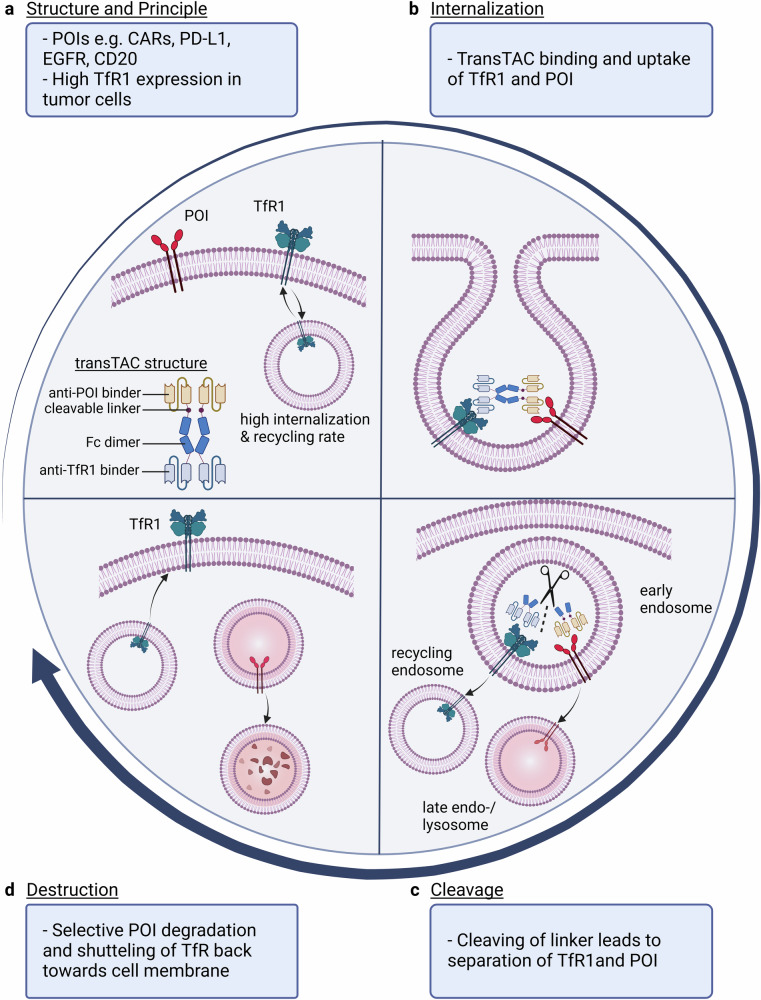
Selective protein degradation using transferrin receptor targeting chimeras (transTACs). **a** The developed structure features anti-POI binding domains and a bivalent anti-transferrin receptor 1 (TfR1) binding domain connected via a cathepsin B-cleavable linker. TfR1 shows an elevated expression level on tumor cells in comparison to normal cells and has a high internalization and recycling rate. The tested protein of interests (POIs) in this study are chimeric antigen receptors (CARs), Programmed Cell Death 1 Ligand 1 (PD-L1), Epidermal Growth Factor Receptor (EGFR) and cluster of differentiation (CD)20. **b** Binding of the POI and TfR1 by the transTACs allows for the internalization of both. **c** Upon cleavage of the transTAC, TfR1 enters the recycling endosome whereas the POI is degraded in the endolysosomal pathway. **d** Via the recycling pathway, TfR1 is shuttled back to the cell membrane. Created with BioRender.com

The therapeutic concept of the study is aiming at the targeted extracellular membrane protein degradation in cancer cells using a newly designed heterobispecific antibody chimera, which is carrying anti-POI binding as well as bivalent anti-transferrin receptor 1 (TfR1) binding domains connected via cathepsin B-cleavable Fc dimers. This cleavable feature is the essential novel component to generate selective lysosomal degradation of the POI while the TfR1 is being recycled towards the cell membrane. The authors name their novel degrading molecule “transferrin receptor targeting chimeras” (transTACs). The targeting concept is based on the long-known fact that cancer cells are highly depending on sufficient iron supply and uptake to ensure proper cell metabolism and growth. The functional consequence is that tumors overexpress TfR1, as shown by the authors and many others.^4^ Thus, exploiting the overexpressed TfR1 on cancer cells to allow targeting of the POI represents a smart tool to achieve target internalization and degradation (Fig. 1).

The great challenge in this approach was to use the TfR1 internalization pathway on one hand while avoiding the entry of the POI into the endosomal TfR1 recycling on the other, which would otherwise prevent POI degradation. This led the authors to the idea of splitting the endosomal and lysosomal routes by using the cathepsin B-cleavable moiety leaving the TfR1-bound part of the transTAC in the endosomal recycling path and guiding the POI bound part towards endolysosomal degradation. In fact, the use of recycling membrane proteins for POI degradation is not novel per se, as shown by e.g. bispecific antibodies which facilitate integrin-mediated lysosomal degradation. However, novelty here comes from their approach to use the TfR1 receptor because of its high internalization rate as well as the intriguing design of using the cathepsin B-sensitive feature within the transTAC. The highly optimized transTACs (with variations in the binding domains and linkers) were first designed to bind to TfR1 and CD19 chimeric antigen receptors (CARs) expressed on Jurkat or HeLa cancer cells and led to an up to 80% POI degradation. The use of such transTACs is of therapeutic relevance, since CAR-targeting can help control CAR-T cell activities and CAR-associated side effects in cancer immunotherapies. Therein, this approach represents an elegant alternative to known genetic switch (suicide switch) control of CAR-T cells, avoiding the extra efforts of genetic engineering of such CAR-T cells before their therapeutic use in patients.

Of note, the study demonstrated the use of the new transTACs as genetic engineered building blocks by which targeting of PD-L1 on MDA-MB-231 breast cancer cells or CD20 on B-cells is possible, efficiently eliminating up to 85% or 95% of these POIs, respectively. This points to the great potential and broad use of the transTACs for immunotherapies providing more advanced means to support such therapies in cancer patients. In addition, the kinetic of transTAC action revealed the high velocity of POI internalization and degradation, supporting the effectiveness of this novel approach.

The attractiveness of the transTAC platform is based on its ability to easily extend the POI target scope, shown for the epidermal growth factor receptor (EGFR). EGFR is overexpressed in many cancer types and contributes to therapy resistance mechanisms in cancer.^5^ Although first- and second-generation tyrosine kinase inhibitors (TKI) are routinely used in the clinic to treat EGFR overexpressing cancers, emergence of TKI resistance via EGFR mutations is a severe problem. The study convincingly shows that EGFR-targeting transTACs efficiently trigger EGFR degradation of varying mutational status even more effective than the respective TKI (afatinib, gefitinib or osimertinib) based targeted therapies, as shown in a non-small cell lung cancer (NSCLC) model and in in vivo testing, demonstrating proper tumor targeting and efficient tumor growth inhibition.

In conclusion, the growing efforts to improve cancer therapies by POI degradation have led to tremendous achievements in the generation of such therapeutic concepts. Along this line, this study highlights how more targeted and more efficient POI degradation can be achieved. The transTAC platform allows—as a modular building block system—the attack of numerous different POI targets of therapeutic relevance on cancer cells, with high POI degradation efficacy and turnover velocity of the TfR docking molecule. The novel design of the transTACs represents an innovative therapeutic strategy with significant impact on targeted cancer treatment. The attractiveness of the approach comes from the fact that transTACs are flexible in targeting and are not limited to a small set of POIs, enabling a quick and easily adaptable therapy for heterogeneous cancer disease.
